# Determinants of pneumonia among 2–59 months old children at Debre Markos referral hospital, Northwest Ethiopia: a case-control study

**DOI:** 10.1186/s12890-019-0908-5

**Published:** 2019-08-13

**Authors:** Sefinew Getaneh, Girma Alem, Maru Meseret, Yihun Miskir, Tilahun Tewabe, Gebeyaw Molla, Yihalem Abebe Belay

**Affiliations:** 1 Debre Markos Referral Hospital, Debre Markos, Ethiopia; 2 Collegeof Health Sciences, Debre Markos University, Debre Markos, Ethiopia; 3 Collegeof Medicine and Health Science, Bahir Dar University, Bahir Dar, Ethiopia; 4 Ethiopian Public Health Institute, Addis Ababa, Ethiopia

**Keywords:** Determinants, Pneumonia, Under five children, Referral hospital, Debre Markos

## Abstract

**Background:**

Pneumonia is a significant public health problem globally. The early identification and management of the determinants of pneumonia demands clear evidence. But, there is a limited data on this issue in the current study area. Thus, this study aimed to identify the determinants of pneumonia among 2–59 months old children at Debre Markos Referral Hospital, Northwest Ethiopia.

**Methods:**

A Hospital based unmatched case-control study was conducted among 334 (167 Cases and 167 Controls) children at Debre Markos Referral Hospital from February 1 to March 30, 2018. Consecutive sampling technique was employed and data were collected with a pre-tested interviewer administered questionnaire. Data were entered into Epi-Data version 4.2, and analyzed using SPSS version 25 software. Bi-variable and multi-variable logistic regression analyses were fitted. Variables having *p-value* < 0.05 were considered as statistically significant.

**Results:**

A total of 328(164 cases and 164 controls) 2–59 months old children were included in this study. Not opening windows daily [AOR:6.15(2.55,14.83)], household near to the street [AOR:4.23(1.56,11.44)], child care by the house workers and relatives [AOR:2.97 (1.11,7.93)], using only water for hand washing before child feeding [AOR:3.81 (1.51, 9.66)], mixed feeding practice from birth to six months [AOR: 7.62 (2.97, 19.55)], having upper respiratory tract infection in the last 2 weeks for the child [AOR: 5.33 (2.16, 13.19)] and children with history of co- residence with URTI family [AOR: 6.17 (2.36,16.15)] were found to be determinants of pneumonia.

**Conclusions:**

The main contributing factors for pneumonia in this study are preventable with no or minimal cost. Therefore, we recommend appropriate and adequate health education regarding pneumonia prevention and control.

## Background

Pneumonia remains the leading cause of death in children under five years old worldwide. In 2016, pneumonia accounts for 15.6 and 17% of all deaths in this age group globally and in Sub-Saharan Africa respectively [1]. Most of these deaths occurred in developing countries where access to care is limited and interventions that have improved care in developed countries are scarce [2]. In Ethiopia, pneumonia contributes for 16.4% of all deaths of children under five years of age more than diarrhea, malaria, AIDS and measles combined [1]. The WHO/UNICEF developed the integrated Global Action Plan for Pneumonia and Diarrhea (GAPPD) in 2013 to end preventable child deaths from Pneumonia and Diarrhea by 2025 [3]. In addition, the Federal Ministry of Health of Ethiopia (EFMOH) has incorporated pneumococcal conjugate vaccine in its expanded program on immunization in 2011 to prevent against the severe forms of pneumococcal disease in childhood [4]. Despite the global and government efforts, pneumonia in Ethiopia remains a considerable problem.

Beyond death, pneumonia in children could result in a substantial economic burden in the affected society. This is mainly related to the costs incurred during diagnosis, outpatient and inpatient treatment of cases at health facilities [5–9]. In Ethiopia, pneumonia leads affected households to be impoverished due to medical payments [10, 11].

Previous studies indicated that some of the most common risk factors for pneumonia include lack of exclusive breastfeeding, indoor air pollution, parental cigarette smoking, malnutrition, common co- morbid conditions, living in crowded house, keeping domestic animals inside the main house, using charcoal for cooking, advanced maternal age, previous upper respiratory tract infection, more than four family members, lack of zinc supplementation, absence of separate kitchen, absence of window in the kitchen, age of child, father’s education, child cared by housekeeper and their relatives, history of diarrhea in child, house near to the street and household history of acute lower respiratory infection [12–20].

The contributing factors need be studied so as to better inform and educate the policy makers, programmers, implementers and the general population about the problem. There is limited evidence regarding the determinants of pneumonia in the current study area. Therefore, this study aimed to identify the determinants of pneumonia among 2–59 months old children at Debre Markos referral Hospital, Northwest Ethiopia.

## Methods

### Study design, area and period

A Hospital based unmatched case-control study design was employed at Debre Markos Referral Hospital, Amhara National Regional State, Northwest Ethiopia. The hospital offers a full range of health care services including child care. It is expected to serve a population of 2,153,937.The hospital has 193 beds used for inpatient services [21]. The study was conducted from February 1 to March 30, 2018.

### Population and eligibility criteria

The source population was all 2–59 months old outpatient department (OPD) visitors in Debre Markos Referral Hospital. A case was defined as a child of age 2–59 months old who has attended in the hospital with a diagnosis of pneumonia within the study period. Diagnosis of pneumonia was determined by the assigned physician using the Federal Democratic Republic of Ethiopia Ministry of Health Integrated Management of Newborn and Childhood Illness (IMNCI) guideline [22] adopted from WHO. A control was defined as a child 2–59 months old who has attended in the Hospital with a diagnosis of non-pneumonia case in the study period. All 2–59 months old children who have attended the OPD of the hospital were included in the study. However, mothers or caregivers of children who were having hearing impairments or unable to talk were excluded from the study.

### Sample size determination and sampling procedure

A sample size of 334 (167 cases and167 controls) was determined using Kelsey et al. formula [23] using the Epi-Info Version 3.5.1 software.

#### Assumptions

Assuming two sided confidence level (CI) = 95%, Power = 80%, ratio of controls to cases =1:1 and taking history of parental smoking as a main predictor of Pneumonia with the percent of controls exposed 19.57% and Odds Ratio 2.1 from a case control study in Southwest Ethiopia [16] and an estimated non-response rate of 10%.

Both cases and their controls were selected by consecutive sampling technique from the under-five OPD visitors (aged 2-59 months old) until the required sample was reached. For every case of pneumonia, one control before or after the case was taken. Finally, interviewing of the mothers/care givers was carried out for both controls and cases.

### Data collection process and instrument

Data were collected using a pretested-interviewer administered structured questionnaire. The questionnaire was prepared first in English and translated in to Amharic, then back to English to check for its consistency. The Amharic version of the questionnaire was used to collect the data. Primary data were collected through face to face interview of the mother/care taker. Information regarding patient’s diagnosis based on assigned physician, patient’s weight/height, weight/age, patient’s malnutrition status (malnourished or not), HIV tested or not, and patient’s sero status (reactive or non-reactive) were collected from patient’s medical records (patient’s charts and registers). Two Nurses (first degree holder) at the hospital collected the data, and one General Practitioner supervised the whole data collection process. The data collectors and supervisor were trained for two days on the study objective and data collection process. The questionnaire was pretested on 5% of the sample size at Finoteselam Hospital, and amendments on the questionnaire were made accordingly. Intensive supervision was done by the supervisor and principal investigator throughout the data collection period.

### Study variables

The dependent variable in this study was Pneumonia. The independent variables were: Socio demographic factors (age of child and mother, sex of child, occupation of mother and father, educational status of mother and father, family size, marital status, age of the mother, residence and ethnicity); Home based and behavioral factors (presence of separated kitchen or not, household near to street, ventilation status of the house, number of windows and opening status, presence of cigarette smoker in the house); Nutritional factors, co- morbidities and care practice (breast feeding status of the child, vitamin A supplementation status, vaccination status of the child appropriate for age, HIV status of the child, history of diarrhea, malnutrition, URTI and co residence with URTI family member).

### Data processing and analysis

The data were checked for completeness and consistencies. Data were also cleaned, coded and entered into Epi-Data software version 4.2, then exported to SPSS version 25 statistical package for analysis. The crude odds ratios with 95% confidence interval were estimated in the unadjusted logistic regression analysis to assess the association between each independent variable and the outcome variable. Variables with *p*-value < 0.25 in the unadjusted logistic regression analysis were considered in the adjusted logistic regression analysis. The Hosmer-Lemeshow goodness-of-fit with enter procedure was used to test for model fitness. Adjusted odds ratios with 95% confidence interval were estimated to assess the strength of the association, and variables with *p-value* < 0.05 were considered significant factors.

### Ethical considerations

Ethical clearance was obtained from Ethical Review Committee (ERC) of Debre Markos University, College of Health Sciences. Permission to conduct the study was also obtained from Debre Markos Referral Hospital administrators before data collection. Written consent was obtained from mothers after they were informed about the study objectives, expected outcomes, benefits and the risks associated with it. Confidentiality and privacy of every respondent’s information were ensured.

## Results

### Socio-demographic characteristics of the study participants

Out of 334 (167 cases and 167 controls) children who approached for interview, a total of 328 (164 cases and 164 controls) were included resulting in a response rate of 98.2%. The mean age of the participants was 25.5 months with a range of 2 to 59 months old. The mean age of cases and controls were 19.7 and 30.8 months respectively. More than 80% of mothers both in cases and controls were above 24 years old. About 94 (57.3%) of the cases and 109 (66.5%) of the controls were residents in the urban areas (Table 1).

**Table 1:** Socio-demographic characteristics of participants in Debre Markos Referral Hospital, Northwest Ethiopia, 2018 (*n* = 164 cases & 164 controls)

Variable	Category	Case	Control
		N (%)	N (%)
Sex of child	Male	98 (59.8)	89 (54.3)
	Female	66 (40.2)	75 (45.7)
Age of child (months)	2–11	63 (38.4)	27 (16.5)
	> 11 to ≤23	44 (26.8)	31 (18.9)
	> 23 to59	57 (34.8)	106 (64.6)
Age of mother (years)	≤24	27 (16.5)	24 (14.6)
	> 24 to34	94 (57.3)	104 (63.4)
	≥35	43 (26.2)	36 (22.0)
Marital status of parents	Married	138 (84.1)	155 (94.5)
	Divorced	7 (4.3)	4 (2.4)
	Separated	19 (11.6)	5 (3.0)
Mother’s educational level	Not read and write	72 (43.9%)	55 (33.5%)
	Primary education	36 (21.9%)	41 (25%)
	Secondary education	29 (17.7%)	18 (10.9%)
	Diploma	21 (12.5%)	33 (20.1%)
	Degree and above	6 (3.6%)	17 (10.4%)
Father’s educational level	Not read and write	59 (36.0)	45 (27.4)
	Primary education	32 (19.5)	37 (22.6)
	Secondary education	30 (18.3)	15 (9.1)
	Diploma	18 (11.0)	19 (11.6)
	Degree and above	25 (15.2)	48 (29.3)
Mother’s occupation	Farmer	74 (45.1)	50 (30.5)
	house wife	42 (25.1)	41 (25.0)
	civil servant	26 (15.9)	42 (25.6)
	Merchant	21 (12.8)	30 (18.3)
	Others	1 (0.6)	1 (0.6)
Father’s occupation	Farmer	74 (45.1)	54 (32.9)
	Daily laborer	13 (7.9)	9 (5.5)
	Civil servant	46 (28.0)	66 (40.2)
	Merchant	31 (18.9)	35 (21,3)
Family member	≤4	72 (43.9)	96 (58.5)
	≥5	92 (56.1)	68 (41.5)
Residence	Urban	94 (57.3)	109 (66.5)
	Rural	70 (42.7)	55 (33.5)

### Home based and behavioral factors related to pneumonia

The houses for most of the cases (95.7%) and the controls (97.0%) had windows. About 35 (22.3%) of cases and 141(88.7%) of controls had windows opened daily. Fifty two (42.1%) of cases and sixteen (9.8%) of controls had a kitchen attached to the living room. About 82(50.0%) of cases and 31(18.9%) of controls had only water for hand washing. Only 6(3.7%) of cases and 1(0.6%) of controls had cigarette smoking family member (Table 2).

**Table 2:** House characterstices of participantsin Debre Markos Referral Hospital, Northwest Ethiopia, 2018 (*n* = 164 cases and 164 controls)

Variables	Category		Case	Control
			N (%)	N (%)
Does your house have window?	Yes		157 (95.7)	159 (97.0)
	No		7 (4.3)	5 (3.0)
Number of windows (*n* = 326; 157 for cases and 159 for controls)	Two and less		122 (77.7)	105 (66.0)
	Three and above		35 (22.3)	54 (34.0)
Windows daily opening (*n* = 326; 157 for cases and 159 for control)	Yes		35 (22.3)	141 (88.7)
	No		122 (77.7)	18 (11.3)
Household is near the street	Yes		53 (32.3)	15 (9.1)
	No		111 (67.7)	149 (90.9)
Fuel material mostly used for cooking	Electric	Yes	39 (23.8)	69 (42.1)
		No	125 (76.2)	95 (57.9)
	Charcoal	Yes	42 (25.6)	58 (35.4)
		No	122 (74.4)	106 (64.6)
	Kerosene	Yes	4 (2.4)	5 (3.0)
		No	160 (97.6)	159 (97.0)
	Wood	Yes	110 (67.1)	71 (48.2)
		No	54 (32.9)	76 (46.3)
Mainly used cooking room	In the living room		10 (6.7)	10 (6.1)
	Kitchen attached to living room		52 (42.1)	16 (9.8)
	In the separate kitchen		102 (51.2)	138 (84.1)
Child stays during cooking	Hold on my back		57 (34.8)	30 (18.3
	Out cooking room		107 (65.2)	134 (81.7)
Who give care for child most of the time?	Parents		119 (72.6)	141 (86.0)
	House worker and relatives		45 (27.6)	23 (14.0)
Cigarette smoking family member	Yes		6 (3.7)	1 (0.6)
	No		158 (96.3)	163 (99.4)
If yes, who smokes?	Father		6 (100)	0 (0)
	Other family member		0 (0)	1 (100)
Hand washing practice before child feeding	Only water		82 (50.0)	31 (18.9)
	Water and ash		3 (1.8)	10 (6.1)
	Water and soap		79 (48.2)	123 (75.0)
Do you have domestic animals?	Yes		75 (45.7)	58 (35.4)
	No		89 (54.3)	106 (64.6)
Are animals kept in the same house?	Yes		33 (44.0)	13 (22..4)
	No		42 (56.0)	45 (77.6)

### Co-morbidity related factors to pneumonia

About 152(92.7%) of cases tested for HIV and 3 (2%) of them were sero-positive while from 73(44.5%) controls tested for HIV, 2(2.7%) of them were sero-positive. Ten (6.1%) of cases and 11(6.7%) of controls had malnutrition. About 123(37.5%) of study participants, 107(65.2%) cases and 16(9.8%) controls had history of URTI for the last two weeks duration prior to the data collection period (Table 3).

**Table 3:** Different morbidities related to pneumonia among 2–59 months old children in Debre Markos Referral Hospital, Northwest Ethiopia, 2018 (*n* = 164 cases and 164 controls)

Factors	Category	Case	Control
		N (%)	N (%)
Pneumonia in the family in the last 2wks?	Yes	10 (6.1)	6 (3.7)
	No	154 (93.9)	158 (96.3)
Child treated pneumonia in 2 weeks	Yes	9 (5.5)	4 (2.4)
	No	155 (94.5)	160 (97.5)
Asthma for the child	Yes	8 (4.9)	4 (2.4)
	No	156 (95.1)	160 (97.6)
Upper respiratory tract infection in the past 2 weeks for the child	Yes	107 (65.2)	16 (9.8)
	No	57 (34.8)	148 (91.2)
Upper respiratory tract infection in the past 2 weeks in the family	Yes	114 (69.5)	12 (7.3)
	No	50 (30.5)	152 (92.7)
Malnutrition for the child	Yes	10 (6.1)	11 (6.7)
	No	154 (93.9)	153 (93.3)
Diarrhea for the child	Yes	37 (22.6)	18 (11.0)
	No	127 (77.4)	146 (89.0)
Family member Tb infection history	Yes	9 (5.5)	2 (1.2)
	No	155 (94.5)	162 (98.)
Child Tb infection history	Yes	1 (0.6)	1 (0.6)
	No	163 (99.4)	163 (99.4)
First visit for sick baby	Traditional healer	14 (8.5)	3 (1.8)
	Health facility	150 (91.5)	161 (98.2)
HIV tested	Yes	152 (92.7)	73 (44.5)
	No	12(.3)	91 (55.5)
If yes, test result	Non-reactive	149 (98.0)	71 (97.5)
	Reactive	3 (2.0)	2 (2.7)

### Nutritional related factors associated to pneumonia

As shown in Table 4, about 66(40.2%) of cases and 148(90.2%) of controls were breastfed exclusively. About 137(83.5%) of cases and 151(92.1%) of controls were vaccinated.

**Table 4:** Nutritional related to pneumonia in Debre Markos Referral Hospital, Northwest Ethiopia, 2018 (*n* = 164 cases and 164 controls)

Variables	Category	Case	Control
		N (%)	N (%)
Breast feeding practice (birth to 6 months)	Exclusive breast feeding	66 (40.2)	148 (90.2)
	Mixed	98 (59.8)	16 (9.8)
Weight for height (WFH)	Wasted	22 (13.4)	14 (8.5)
	Normal	142 (86.6)	150 (91.5)
Weight for age (WFA)	Under weight	21 (12.8)	13 (7.9)
	Normal	143 (87.8)	151 (92.1)
Vitamin A Supplementation	Yes	81 (37.5)	42 (37.5)
	No	135 (62.5)	70 (62.5)
Vaccination status appropriate for age	Yes	137 (83.5)	151 (92.1)
	No	27 (16.5)	13 (7.9)

### Bi-variable and multi-variable analysis result

In the unadjusted analysis, a total of 12 variables were found to full fill the assumption of logistic regression at *p*-value of less than 0.25 and nominated for further analysis in the adjusted analysis. By using backward logistic regression method, 7 variables (windows daily opening status, household near to street, child care giver most of the time, hand washing material used before child feeding, birth to 6 months breast feeding practice, URTI in the last 2 weeks for the child and a child with history of co- residency with family member who had URTI) had showed significant association with pneumonia at *p*- value less than 0.05.

The odds of developing pneumonia among children with no windows daily opening was 6.15 times more as compared to those children with windows daily opening [AOR:6.15 (2.55, 14.83)].

The probability of having pneumonia in children with a house near to the street was 4.23 times more as compared to those children with a house not near to the street [AOR:4.23 (1.56,11.44)].

Those children who got child care mostly from the house workers and relatives were 2.97 times more likely to develop pneumonia than those children who mostly got child care from their parents [AOR:2.97 (1.11,7.93)].

The odds of developing pneumonia among children with hand washing before feeding using water only was 3.80 more as compared to those children with hand washing using both water and soap [AOR:3.81 (1.51, 9.66)].

Those children who had mixed feeding were 7.62 more likely to develop pneumonia than those children with exclusive breast feeding from birth to six months [AOR: 7.62(2.97, 19.55)].

The likelihood of developing pneumonia in children who co-reside with a person with URTI was 6.17 times more as compared to their counterparts [6.17 (2.36,16.15)].

Those children who had URTI in the last 2 weeks prior to the study period were 5.33 times more likely to have pneumonia than their counterparts [AOR:5.33 (2.16,13.19)] (Table 5).

**Table 5:** Determinants of pneumonia among 2–59 months old children in Debre Markos referral hospital, Northwest Ethiopia, 2018 (*n* = 164 cases and164 controls)

Variables	Category	COR (95% CI)	AOR (95% CI)
Age of child (months)	2–11	4.34 (2.59, 7.55)	2.35 (0.83, 6.71)
	> 11 to ≤ 23	2.64 (1.51, 4.63)	1.76 (0.66, 4.71)
	> 23 to 59	1	1
Mother’s educational level	Can’t read and write	3.71 (1.37, 10.02)	7.16 (0.77, 66.75)
	Primary	2.48 (0.88, 6.98)	4.99 (0.66, 37.55)
	Secondary	4.56 (1.52, 13.30)	2.49 (0.35, 17.75)
	Diploma	1.80 (0.61, 5.31)	4.65 (0.68, 31.80)
	Degree and above	1	1
Family member	≤4	1	1
	≥5	1.80 (1.16, 2.79)	2.13 (0.93, 4.98)
Windows daily opening	Yes	1	1
	No	27.31 (14.70, 50.70)	6.15 (2.55, 14.83)
Mainly used cooking room	In the living room	1.35 (0.54, 3.30)	0.97 (0.19, 5.11)
	Attached to living room	4.39 (2.38, 8.14)	3.35 (0.96, 11.03)
	Separate kitchen	1	1
Mostly child care giver	Parents	1	1
	House worker and relatives	2.32 (1.33, 4.05)	2.97 (1.11, 7.93)
Hand wash before child feeding	Only water	4.12 (2.50, 6.80)	3.81 (1.51, 9.66)
	Water and ash	0.47 (0.13, 1.75)	0.38 (0.03, 4.84)
	Water and soap	1	1
Birth to 6 months breast feeding	Exclusive breast feeding	1	1
	Mixed	13.50 (5.30, 25.10)	7.62 (2.97, 19.55)
House near the street	Yes	4.74 (2.54, 8.85)	4.23 (1.56, 11.44)
	No	1	1
Vaccination appropriate for age	Yes	1	1
	No	0.44 (0.21, 0.88)	0.50 (0.08, 3.04)
URTI in the last 2 weeks	Yes	17.36 (9.45, 31.80)	5.33 (2.16, 13.19)
	No	1	1
Co-residence with URTI person	Yes	28.8 (14.7, 56.73)	6.20 (2.36, 16.15)
	No	1	1

Note: variables were included in the adjusted model based on a *p*-value of < 0.25 in the unadjusted model

## Discussion

The main aim of this study was to investigate the determinants of pneumonia among 2–59 months old children at Debre Markos referral Hospital in Northwest Ethiopia.

In the current study, respondents who did not open their house windows daily were at higher risk of developing pneumonia than their counter parts. It is consistent with other studies which reported that poor indoor ventilation was the risk factor associated with pneumonia, and ventilation is poor in a house without window or unopened windows daily, house attached with kitchen or used for kitchen and living [12, 18, 24]. This could be due to the fact that dust particles stay to the house resulting indoor air pollution and may cause frequent respiratory problems which can progress to pneumonia especially in children as their immunity is under developed. Healthy homes require sufficient light, especially natural light in the form of ultraviolet light which can kill germs, bacteria, viruses, and fungi that can cause infection, allergies, asthma or other diseases [25].

In relation to child care giver’s relationship with the child, children who got care by the house worker and relatives were at higher odds of developing pneumonia as compared to children who cared by their parents. This result is in line with a study conducted in Oromia Zone of Amhara region [14] where the study showed children cared by house keeper and their relatives were 2.79 times more likely to develop pneumonia as compared to children cared by their parents. This might be due to the fact that house workers and relatives may not be responsible for child caring as their parents such as keeping child’s self-hygiene, feeding the child on time and other child care activities.

In the present study, those children from a house near to the street were at increased odds of developing pneumonia than those who were not lived near the street. This result is consistent with a study conducted in China which found that living near traffic-related facilities was likely a risk factor for childhood pneumonia among urban children [13]. This could be explained by the fact that in areas where street are grave and dusty, the probability of developing respiratory related diseases is high. Streets in small towns of Ethiopia (including Debre Markos referral hospital catchment area- the current study area) are usually dusty especially during winter season which might predispose children to pneumonia.

Regards to hand washing practice before feeding a child, those respondents who had used water only were at higher odds of developing pneumonia than those who had used water and soap. The World Health Organization recommends children and care takers should wash their hands at critical times throughout the day with water and soap to decrease the risk of exposure to bacteria and other microbial agents which can cause pneumonia [26].

In relation to breastfeeding practice in the first six months of life, the odds of developing pneumonia in children who were on mixed breastfeeding practice was higher as compared to children who were exclusively breastfed during their birth to six months old duration. This is consistent with previous studies [16, 17, 27, 28]. This could be due to the fact that children who were not exclusively breastfed were more prone to infections like pneumonia as a result of sub optimal feeding practice. WHO recommends that the infant should only receive breast milk without any additional food or drink, not even water since breast milk is the ideal food for the healthy growth and development of infant [29].

This study also revealed that children who had URTI in the last 2 weeks were more likely to develop pneumonia than those who did not have URTI. It is consistent with a study conducted in Southwest Ethiopia [16] which reported that children with previous upper respiratory tract infection were 5.2 times more likely to develop pneumonia as compared to their counterparts. Additionally, this study found that having history of co-residence with a family member who had URTI was strongly associated with developing pneumonia which is in line with other recent studies [14, 19]. The possible explanation might be due to the fact that URTIs are highly communicable and can be transferred through air born, direct, and indirect contacts. Then, once the infection established, it permits invasion of the lung by microorganisms that trigger the immune response and produce inflammation [30, 31].

## Conclusions

In the present study, factors related with pneumonia were windows daily opening status, household near to street, child care giver most of the time, hand washing material used before child feeding, birth to 6 months breast feeding practice, URTI in the last 2 weeks for the child and a child with history of co- residency with family member who had URTI. These determinants are preventable with no or minimal cost. Therefore, we recommend appropriate and adequate health education regarding pneumonia prevention and control.

## Data Availability

The data of this study cannot be shared publically due to the presence of sensitive (confidential) participants’ information.

## Abbreviations

(EFMOH): Federal Ministry of Health of Ethiopia
AIDS: Acquired Immune Deficiency Syndrome
AOR: Adjusted Odd Ratio
COR: Crude Odd Ratio
GAPPD: Global Action Plan for Pneumonia and Diarrhea
HIV: Human Immune Deficiency Virus
OPD: Out-Patient Department
OR: Odd ratio
SPSS: Statistical Package for Social Sciences
UNICEF: United Nations Children’s Fund
URTI: Upper Respiratory Tract Infection
WHO: World Health Organization
